# The effect of methylphenidate on neurofibromatosis type 1: a randomised, double-blind, placebo-controlled, crossover trial

**DOI:** 10.1186/s13023-014-0142-4

**Published:** 2014-09-10

**Authors:** Laurence Lion-François, François Gueyffier, Catherine Mercier, Daniel Gérard, Vania Herbillon, Isabelle Kemlin, Diana Rodriguez, Tiphanie Ginhoux, Emeline Peyric, Virginie Coutinho, Valentine Bréant, Vincent des Portes, Stéphane Pinson, Patrick Combemale, Behrouz Kassaï, et Réseau NF1 Rhône Alpes Auvergne-France

**Affiliations:** Department of Pediatric Neurology, Mother & Child Hospital, 59, boulevard Pinel, 69677 Bron Lyon, France; Departement of Clinical Pharmacology & Clinical Trials, UMR 5558, Univ Lyon, F-69000 France, CHU Lyon, Hop L Pradel, F-69000 Lyon, France; Hospices Civils de Lyon, Service de Biostatistique, F-69003 Lyon, France; Université de Lyon, F-69000 Lyon, France; Université Lyon 1, F-69100, Villeurbanne France; CNRS, UMR 5558, Laboratoire de Biométrie et Biologie Evolutive, Equipe Biotatistique-Santé, F-69100 Villeurbanne, France; Department of Child Psychiatry, Groupement Hospitalier Est, CHU Lyon, Lyon, F-69000 France; AP-HP, Department of Pediatric Neurology and Neurofibromatosis Reference Centre, Armand Trousseau Hospital, Paris University Hospital East, F-75012 Paris, France; UPMC University Paris 06, F-75012 Paris, France; Inserm, CIC1407, University Lyon, Lyon, F-69000 France; CHU Lyon, Hop L Pradel, Departement of Clinical Pharmacology & Clinical Trials, EPICIME, Lyon, F-69000 France; Hospices Civils de Lyon, Pharmacy, Groupement Hospitalier Est, CHU Lyon, F-69000 France; Université de Lyon, F-69008 Lyon, France; Human Genetics Laboratory, Hôpital Edouard Herriot, F-69000 Lyon, France; Réseau NF1 Rhône Alpes Auvergne IHOP, Lyon, France; Service de neurologie pédiatrique, Hôpital Femme, Mère, Enfant, 59, boulevard Pinel, 69677 Bron, Cedex France

**Keywords:** Neurofibromatosis, Randomised controlled trials, Methylphenidate, Attention deficit hyperactivity disorder

## Abstract

**Background:**

Neurofibromatosis type 1 (NF1) is an autosomal dominant disorder with an estimated prevalence of about 1/3000, independent of ethnicity, race, or gender. Attention Deficit Hyperactivity like Disorder (ADHD)-like characteristics are often reported in patients with NF1. We hypothesised that learning disabilities in NF1 children were related to ADHD symptoms. Treatment with methylphenidate (MPD) has improved learning disabilities in ADHD by acting on neurotransmitters. Our objective was to evaluate its efficacy on ADHD-like symptoms in neurofibromatosis type 1 children (7–12 years).

**Methods:**

This was a randomised, double blind, placebo controlled, and crossover trial comparing 0.5 to 0.8 mg/kg/d of MPD as it is indicated for ADHD to placebo in NF1 children with ADHD-like symptoms. Children aged 7 to 12 years were eligible when their IQ was between 80 and 120. The total follow-up was 9 weeks including 4 weeks for each period and 1 week wash out. Fifty subjects (25 for each period) were required for testing the primary study hypothesis. The main outcome was an improvement in scores on the simplified Conners’ Parent Rating Scale.

**Results:**

Thirty-nine patients were included between April 2004 and December 2010. Twenty participants received MPD and 19 placebo during the first period. They all completed the trial. MPD decreased the simplified Conners by 3.9 points (±1.1, p = 0. 0003).

**Conclusions:**

This is the first randomised controlled trial showing the short-term benefit of MPD on simplified Conners scores in NF1 children.

**Trial registration:**

ClinicalTrials.gov NCT00169611.

## Background

Neurofibromatosis type 1 (NF1), also known as von Recklinghausen’s disease, is an autosomal dominant disorder with an estimated prevalence of about 1/3000 [1]. Caused by mutation in the NF1 gene, this condition is characterised by multiple café-au lait spots, benign neurofibromas and Lisch nodules [2].

The evolution of NF1 is unpredictable and highly variable. It may be undetectable by the untrained eye, lead to mild disfigurement or even life threatening conditions [3]. The physical features of NF1 are well characterised, facilitating the diagnosis after clinical examination.

The main complication of NF1 in childhood is cognitive impairment, which affects quality of life and leads to learning disabilities undermining academic achievement in 30-70% of cases [4–6]. The cognitive disorders in NF1 include memory, switching attention coordination, language, and behavioural disorders [7–9]. NF1 patients have average intelligence quotient (IQ) scores, with a slight decrease in mean IQ [10]. The incidence of mental retardation is 4-8%, which is slightly higher than in the general population [4,10,11].

A high prevalence of ADHD-like characteristics is reported in patients with NF1 [4,9,12–15]. The ADHD-like behaviour in the NF1 population may be a major contributor to academic underachievement in this population [16]. Children with NF1 have difficulties with social interactions, and present a high frequency of internalising features such as anxiety and depression [4]. It is recognised that the cognitive, motor, and social problems are NF1-related disorders, rather than independent comorbid conditions [17].

Medicines used to treat ADHD-like behaviours in NF1 patients have not been evaluated by randomized controlled trials (RCTs). Methylphenidate seems to decrease hyperactivity and increase the attention span and concentration by acting on dopaminergic and noradrenergic neurotransmissions in the central nervous system [18]. Two systematic reviews suggest a favourable risk-benefit profile for MPH [19,20]. Potential risks are headaches, sleeping problems, tiredness, decreased appetite, psychotic symptoms and mood disorders.

It has been suggested that low doses (5 to 15 mg) of methylphenidate (MPD) may improve Test of Variables of Attention (TOVA) and Child Behaviour Checklist (CBCL) scores after one-year of follow-up [14]. Even though MPD has demonstrated its efficacy in reducing ADHD behaviour in children [21], to our knowledge, there have been no reported randomised controlled trials comparing MPD to placebo in NF1 children with school and attention difficulties.

## Methods

### Participants and design

The participants were 7 to 12 years of age with an IQ between 80 and 120, measured using the Wechsler Intelligence Scale for Children (WISC IIIR or WISC IV). A child neurologist, a child psychiatrist and a psychologist enrolled children in two clinical sites, in Lyon and Paris, if they met these inclusion criteria:

1. NF1 diagnosed using two or more of the following National Institutes of Health (NIH) criteria [22]: (1) six or more café au lait macules over 5 mm at the greatest diameter in prepubertal children and over 15 mm in postpubertal children, (2) two or more neurofibromas of any type or one plexiform neurofibroma, (3) freckling in the axillary or inguinal regions, (4) two or more Lisch nodules, (5) optic glioma, (6) a distinctive bone lesion such as sphenoid dysplasia or thinning of long bone cortex with or without pseudarthrosis, or (7) a first-degree relative with NF1 as defined by the above criteria;
2. School difficulties pointed out by parents or teachers;
3. Attention difficulties as defined by anamnesis.

We had the following exclusion criteria:

1. 80 < IQ > 120 measured using the WISC III or WISC IV;
2. Depression;
3. Unwillingness to participate;
4. Patients with NF1 cerebral complications (chiasma tumor, moya-moya, cerebral glioma) detected using MRI;
5. Participation in another interventional study.

The NF1 Attention Study was a randomised, double-blind, placebo-controlled, crossover trial. Total follow-up lasted 9 weeks including 4 weeks for each period and a 1-week wash out period.

The main outcome was the improvement of the simplified Conners’ Parent Rating Scale [23] because it is largely used in children and a French version was available.

The short version comprises 10 questions and provides evaluation of the key areas of inattention, hyperactivity/impulsivity, learning problems, behavioural disorders, anxiety, and peer relations. Specifically developed for detecting hyperactivity, it allows quantifying the intensity of hyperactivity and assessing its various dimensions: hyperactivity, inattention and impulsivity. The score varies from 0 to 30, scores >15 are prognostic of hyperactivity.

The secondary outcomes were the improvement of scores on the Conners’ Teacher Rating Scale. The short version comprises 10 questions provides evaluation of the key areas of (behavioural disorders, hyperactivity/impulsivity, immaturity and passivity). The interpretation of scores is similar to Conner’s Parent Rating Scale.

The Children’s Depression Rating scale (CDRs-R) [24], the Children’s Depression Inventory (CDI) [25], and the State-trait Anxiety Inventory for Children (STAIc) [26] were used in order to monitor anxiety and depression symptoms of participants during the trial.

CDRs-R is a 17-item measure used to determine the severity of depression in children 6–12 years of age. 14 items are based on parent, child and schoolteacher interviews, 3 items from the direct observation of children (depression affects, language time, and hypoactivity). Items are measured on 7 points or 5 points scale. The CDRS is derived from the Hamilton Rating Scale for Depression (HAM-D); a score of 15 on the CDRS is equivalent to a score of 0 on the HAM-D. A score ≥ 40 indicates depression but is not enough to confirm the diagnosis. A score between 40 and 60 characterize light or moderate and scores > 60 severe depression. Score < 30 are normal.

The CDI is adapted from Beck inventory depression to determine the severity of depression in children for children 7 to 17. It’s a psychological assessment that rates the severity of symptoms related to depression and/or dysthymic disorder in children and adolescents, is a 27-item scale that is self-rated and symptom-oriented. Each item is score from 0 to 2. The higher the score, the higher the severity of depression. A score above 15 indicates depression.

The STAIC consists of two 20-item scales that measure state and trait anxiety in children between the ages of 8 and 14. The A-State scale examines the shorter-term state anxiety that is commonly specific to situations and the trait anxiety measures general anxiety. It prompts the child to rate 20 statements from hardly ever true (1point) to often true (3 points). Scores vary from 20 to 60. Score > 34 indicate anxiety.

### Ethical approvals and consent

The study protocol was approved by the ethics committee « Comité de Protection des Personnes dans la Recherche Biomédicale Sud Est II » (file number 2003–042). The study was also registered on ClinicalTrials.gov (NCT00169611). All subjects provided informed consent.

### Sample size and power calculation

Based on a randomised controlled trial by Greenhill et al. [23] and our clinical experience, increasing simplified Conners’ Parent Rating Scale scores by three points was defined as a clinically relevant benefit. To achieve this benefit with a 5% α risk (two-tailed), assuming a within-subject standard deviation of 6.3, 50 subjects (25 for each period) were needed to test the primary study hypothesis with 90% power.

### Randomisation

Patients were enrolled at the Department of Paediatric Neurology (Lyon and Paris Trousseau teaching hospitals). Two physicians, a child neurologist, and a child psychiatrist, asked for children’s assent and informed consent from both parents. The identity of eligible patients was transmitted by the Department of Paediatric Neurology to the Clinical Investigation Centre (CIC) of Lyon and the MPD prescription was faxed to the central pharmacy where a masked randomisation list according to a computer generated randomization allowing concealed allocation was available. Randomisation was performed by a computer generated random number list prepared by the department of biostatistics of the coordination center with no clinical involvement in the trial. The random list was created using SAS (version 8.2) statistical software with a 1:1 allocation using block size of 4.

Patients received their treatment directly from the central pharmacy. Neither patients nor investigators knew which treatment was given.

The CIC and the Department of Biostatistics of Lyon Teaching Hospitals, Lyon, France, conducted data management and statistical analyses. All data were recorder in case report forms and entered by two independent technicians blindly in a central secured database under the responsibility of Clininfo. Site monitoring was carried out by the CIC. A neurofibromatosis association and the regional healthcare network for neurofibromatosis informed potential eligible patients about the trial.

### Intervention

MPD or matched placebo was started at a dose of 0.5 mg/kg/day and increased to 0.8 mg/kg/day if symptoms did not improve after a one-week check-up [13]. There was a one-week washout between the two treatment periods to avoid a carry-over effect due to MPD’s short half-life (2 hours) [24]. Treatment adherence was measured by counting returned pills [25]. Six visits were organized for the inclusion of eligible participants, randomisation, dose-adjustment at week 1, end of first period at week 4, debut of the second period at week 5, the dose-adjustment at week 6, and end of study visit at week 9. Efficacy measures and adverse events were collected by the investigators.

The efficacy of MPD on ADHD disorders has been proven using a minimum dose of 0.3 mg/kg/day. According to the guidelines, the patient should not be given more than 2 or 3 doses of 1 mg/kg/day. Treatment should begin with 5 mg, 2 times per day. The dose should be gradually increased by 5-10 mg per week, without exceeding the maximum dose of 60 mg per day (*source: leaflet from VIDAL*).

### Statistical analysis

The efficacy analyses adhered to the intention-to-treat approach regardless of treatment status at the time of analysis. To compare efficacy between MPD and placebo, we performed a two-factor analysis of variance (ANOVA for repeated measures) on the 2 N measurements with period and treatment factors (MPD versus placebo). Interactions between patient characteristics and treatment effect were also examined. Missing data were replaced only when less than 50% of answers were missing to compute a score. Imputation was performed for each dimension (multidimensional scales) or directly (one-dimensional scale) with the mean for completed items [26]. The global score was then computed as the sum of scores for each dimension (multidimensional scales) or directly (one-dimensional scale).

The results presented in the tables are based on raw data (score variations) but the p-values in the text were calculated using non-parametric ANOVA (ANOVA performed on normal scores from ranks computed with the Van der Waerden method) [27]. Data from each patient were used to estimate the treatment effect. Missing measurements (7/78) were considered as missing by chance, with the same mean as non-missing measurements. None of the patients were excluded from the analysis.

## Results

**Figure 1:**
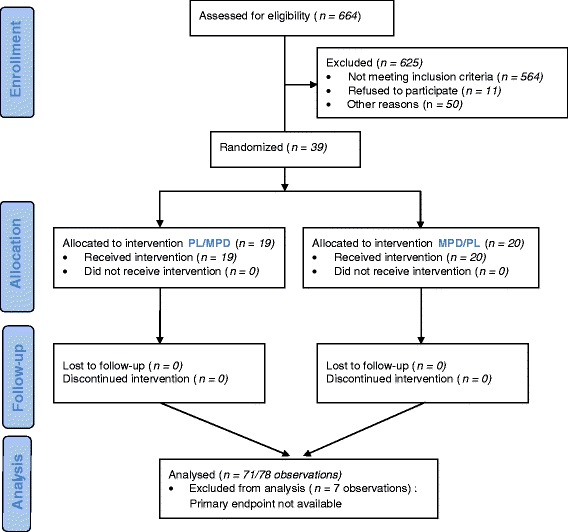
**Participants flow diagram.**

During the recruitment period, out of 664 children screened with NF1 in our registry, 625 were disqualified. This is because 564 did not satisfy inclusion criteria (443 under or over aged, others without attention difficulties, etc.). The parents of 11 children did not give their consent. Thirty-one were not enrolled for other reasons. The reason for non-enrolment is unknown for 19 children (Figure 1). The patient population is described in Table 1. A total of 39 participants, 10 females and 29 males, aged 7.9-12.9 were included from April 2004 to December 2010. The mean age was 9.3 years ± 1.8. The baseline characteristics of the patients did not differ significantly between the two groups, except that those in the MPD/Placebo group had higher IQ scores (p = 0.03). School difficulties in 33% (13/39) of the participants lead to educational consequences. One was in a special class and 12 repeated a grade. Scores on the Conners’ Global Index-Parent (short version) did not differ statistically between groups at baseline (p = 0.37) and neither did the other Conners’ scores (p ≥ 0.12). Based on the Diagnostic and Statistical Manual of Mental Disorders, Fourth Edition (DSM-IV), 18 participants presented ADHD symptoms, 12 only had attention deficit and 2 only had hyperactivity. Seven patients do not present attention deficit or hyperactivity symptoms but remain eligible because they presented school difficulties and attention deficit based on the anamnesis.

**Table 1:** **Demographics and baseline characteristics of the participants**

	**MPD/Placebo**	**Placebo/MPD**
	***(n = 20)***	***(n = 19)***
Age (y)		
Mean*	9.60 +/−2.02	8.98 +/−1.63
Range	7.09-12.99	7.21-11.81
Gender		
Male	16 (80.0%)	13 (68.4%)
Female	4 (20.0%)	6 (31.6%)
Weight (kg)*	29.34 (5.91)	29.04 (6.14)
Height (cm)*	132.50 (10.09)	133.63 (9.15)**
School backwardness^◊^	6 (30.0%)	7 (36.8%)
IQ*	102.0 (12.5)^‡^	94.6 (11.2)^†^
Conners’ global index-Parents*	66.2 (13.9)^‡^	69.8 (16.1)^†^
Conners’ global index-Teacher*	55.9 (8.4)^¥^	59.8 (9.0) ^¥^
Conners’ global index-Short version-Parents*	15.2 (5.2)	13.7 (6.4)
Conners’ global index-Short version-Teacher*	8.1 (4.7)^¤^	11.9 (6.1)**
CDRS-R*	29.9 (7.8)	27.6 (7.4)
CDI*	11.0 (6.5)	10.0 (4.6)
STAI-C 1*	12.9 (7.4)	10.9 (6.5)
STAI-C 2*	15.9 (9.1)	13.3 (7.5)
DSM IV THADA [28]		
Inattention*	6.8 (1.5)	6.9 (2.0)
Hyperactivity/Impulsivity*	5.3 (2.0)	5.8 (3.0)
Hyperactivity*	3.0 (1.6)	3.9 (2.1)
Impulsivity*	2.3 (0.7)	1.9 (1.2)

*Mean +/− SD.

^◊^According to the difference between the child age and the expected age for the school level.

**3 missing values.

^‡^1 missing value.

^†^2 missing values.

^¥^4 missing values.

^¤^5 missing values.

The 39 patients completed the entire 9 weeks study evaluation. Twenty participants received MPD and 19 placebo during the first period. We did not detect any carry-over effect between the two periods (p = 0.41). The flow diagram of patient recruitment and follow-up is presented in Figure 1.

**Figure 2:**
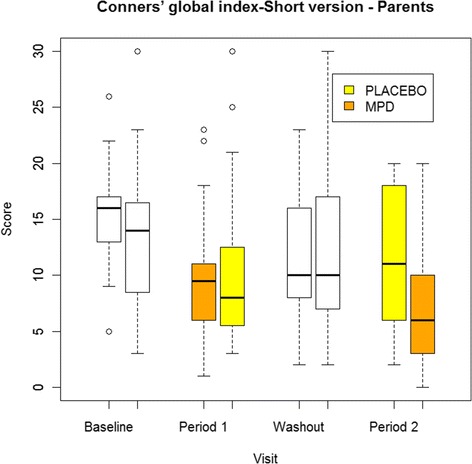
**Evolution of the principal outcome, Conner’s global index short version by treatment group and for each period.**

Simplified Conners’ Parent Rating Scale scores decreased by 3.9 points (±1.1, p = 0. 0003). Results on primary and secondary outcomes are presented in Table 2. In the MPD group, the baseline score (mean ± SD) of 13.8 ± 6.4 was reduced to a mean score of 8.2 ± 5.6 in week 4. The baseline and week 4 mean scores in the placebo group were 12.7 ± 6.9 and 10.8 ± 6.9. (Figure 2).

**Table 2:** **Efficacy of methylphenidate on the simplified Conners’ rating scale and other secondary endpoints**

	**Within-patient difference**		**MPD effect**
	**MPD**	**Placebo**	
Parent’s version (Main endpoint)	−5.7 (−7.3, −4.1)	-1.8 (−3.4, −0.1)	-3.9 (−6.1, −1.7)
Teacher’s version	−3.5 (−5.8, −1.2)	-1.6 (−3.8, 0.6)	-1.9 (−5.0, 1.1)*
CDRs	−2.9 (−5.0, −0.9)	-1.9 (−4.0, 0.2)	-1.1 (−4.0, 1,8)
CDI	−2.1 (−3.9, −0.4)	-1.9 (−3.7, −0.1)	-0.2 (−2.7, 2.3)
STAIc-state	−3.2 (−5.1, −1.2)	-1.9 (−3.8, 0.1)	-1.3 (−4.0, 1.4)
STAIc-trait	−3.1 (−5.2, −1.0)	-2.7 (−4.8, −0.6)	-0.3 (−3.3, 2.6)

*NS, 50% of data were missing.

Mean variation (95% confidence limits).

The effect of MDP on simplified Conners’ Teacher Rating Scale scores cannot be interpreted because half of the data were missing. MDP had no effect on depression scores (CDRs and CDI) or on anxiety scores (STAIC-state and STAIC-trait).

Non-adherence to methylphenidate with no apparent reason was reported for two children. Mean compliance was 75.30% (SD ± 13.56), ranging from 26.42 to 100%; with a median of 77.40% and an upper quartile of 81.17%. The mean dose of MPD prescribed was 16.3 mg/day. Compliance was not evaluated for 11 patients because of missing data (7 in the sequence group MPD/Placebo and 4 in the sequence group Placebo/MPD). When available, compliance was not statistically different between sequence groups (p = 0.66).

Adverse events are reported in Table 3. One serious adverse event was reported in a patient, who had gastrointestinal disorders while taking MPD. The investigator found that this event was not related to the study drug.

**Table 3:** **Adverse events reported in each group**

	**Placebo (%)**	**MPD (%)**	**Total**
Insomnia, Anxiety/Nervousness	2 (16,67)	11 (37,93)	13
Headache	3 (25,00)	3 (10,34)	6
Anorexia/Decreased appetite	0 (0,00)	6 (20,69)	6
Abdominal pain	2 (16,67)	2 (6,90)	4
Abnormal loss of weight	1 (8,33)	1 (3,45)	2
Vomiting/Nausea	0 (0,00)	2 (6,90)	2
Other*	4 (33,33)	4 (13,79)	8
Total	12	29	41

*Tonsillitis, dysphemia, hot flush, enuresis, asthenia, attention deficit, rash.

## Discussion

In our daily clinical practice visiting patients referred for NF1 present a high prevalence of ADHD-like symptoms and academic underachievement. No evidence-based guideline on how to treat these patients is currently available. Our study aimed to answer the question whether our everyday patients with NF1 having ADHD-like characteristics based on the Diagnostic and Statistical Manual of Mental Disorders, Fourth Edition (DSM-IV), could potentially benefit from methylphenidate without being harmed by adverse events such as depression or anxiety disorders.

In our study Conners’ Parent Rating Scale scores decreased by 3.9 points by MPD, suggesting a clinically relevant benefit. Our results are very close to those observed in children with ADHD reported by Greenhill et al. [23]. Because of the high number of missing questionnaires for our secondary outcome, we were not able to show a significant reduction in symptoms, based on results from the teacher version of the 10-item Conners’ Global Index.

After the end of the study, 37 participants continued to take MPD or switched from placebo to MPD. This group had normal IQ. Scores on the CDRS, the CDI, and the STAIC [29] did not increase in NF1 patients before treatment or during the four weeks into the study. MPD did not seem to have an impact on depression and anxiety during our study.

Stimulants are the main drugs used to treat ADHD. Defining the target population [30] and ensuring the safety of stimulants are sources of concern and regularly relayed by the media [31]. A database evaluation of the use of MPD suggests a 2.5-fold increase from 1990 to 1995, but it is not clear if this practice is appropriate [30]. The use of MPD has been limited to patients with neurological disorders because of its side effects such as motor tics, sleep disorders, headaches, decreased appetite, stomach pain, nausea, irritability, seizures [32], and growth suppression [33]. However, it is not certain that MPD is responsible for these adverse events. For instance, the tic rate reported by Schachar et al. was similar in children taking placebo and those treated with MPD [34]. In a recent long-term follow-up study, loss of appetite was the most common adverse effect of MPD [25]. Administration of MPD during or after meals may minimize the influence of anorexia. The correlation between growth suppression and treatment has not yet been established and it is not clear whether drug holidays are necessary, even though they are recommended in France [35]. Drug holidays during summer months have been proposed as “catch up” growth periods [33]. Lastly, a recent study showed that current users of ADHD drugs did not have an increased risk of developing serious cardiovascular events [36].

There are several limitations to this study. We used the French version of the Conners’ Parent Rating Scale because it was the only scale available in French when we started our study. Few questionnaires were received for the teacher version, but their results show also a beneficial trend during the MPD period (−1.9, p = 0.22). Even though another NF1 reference centre in Paris helped recruiting patients, we did not reach our target sample size of 50 patients. The two-year recruitment period planned for our study was not long enough mainly because, MPD could not be prescribed during school holidays because dose adaptation requires close patient monitoring, potential hyperactivity drug-related deaths made parents suspicious of its safety [31] and reluctant to participate, and finally, exclusion criteria were more common than anticipated. We asked an independent data monitoring committee (DMC) comprising a clinical pharmacologist and a biostatistician to advise us on trial recruitment and whether or not we should stop the trial earlier. The DMC did not un-blind the data and suggested extending the recruitment period by one year to include three or four more patients. This would mean 10% more participants and would have potentially increased the power of the study. After these measures were implemented (March 2005), we were able to include 39 of the 50 participants we had originally hoped for. After 54 months extension, the study was stopped because the available pool of patients was already screened for the trial and the pool of newly diagnosed patients would not allow us to terminate the study within an acceptable period of time. However, our final results suggest that the efficacy of MPD was greater than expected. Finally, the duration of the treatment by MPD was only four weeks and the long-term benefit should be assessed adequately.

## Conclusion

MPD showed a short-term benefit on the Conners’ Parent Rating Scale in NF1 children with school difficulties and attention deficit.
